# Supplementary material for: Transport of Alzheimer’s associated amyloid-β catalyzed by P-glycoprotein
Source: PLoS One. 2021 Apr 26;16(4):e0250371. doi: 10.1371/journal.pone.0250371 (PMC8075256; doi:10.1371/journal.pone.0250371)
Supplement: S1 Table — (DOCX) [file pone.0250371.s010.docx]

**S1 Table. Number and Type of Residue Contacts made by Aβ monomers with the Drug Binding Domains of P-gp during TMD simulations.**

|  | **Residue Category and Number of Contacts** | | | |
| --- | --- | --- | --- | --- |
| **Aβ40 (2LFM)** | **Polar** | **Non-Polar** | **Positively-Charged** | **Negatively-Charged** |
| **4KSB (start)** | 13 | 30 | 1 | 0 |
| **3B5X** | 10 | 24 | 2 | 0 |
| **2HYD** | 16 | 27 | 1 | 0 |
| **3B5Z (end)** | 13 | 23 | 1 | 0 |
| **Aβ40 (2M4J)** | **Polar** | **Non-Polar** | **Positively-Charged** | **Negatively-Charged** |
| **4KSB (start)** | 12 | 30 | 2 | 1 |
| **3B5X** | 9 | 26 | 4 | 2 |
| **2HYD** | 18 | 33 | 3 | 3 |
| **3B5Z (end)** | 19 | 30 | 2 | 2 |
| **Aβ42 (1IYT)** | **Polar** | **Non-Polar** | **Positively-Charged** | **Negatively-Charged** |
| **4KSB (start)** | 10 | 35 | 4 | 1 |
| **3B5X** | 9 | 28 | 2 | 4 |
| **2HYD** | 18 | 34 | 2 | 3 |
| **3B5Z (end)** | 13 | 28 | 1 | 2 |

Data are reported as the number of residues in the P-gp Drug Binding Domain with an α-carbon within 3 Å of the Aβ peptide. Residues are classified as Polar (SER, THR, CYS, ASN, GLN, TYR), Non-Polar (GLY, ALA, VAL, LEU, MET, ILE, PHE, PRO, TRP), positively Charged (LYS, ARG, HIS), negatively Charged (GLU, ASP).
