# Supplementary material for: Transport of Alzheimer’s associated amyloid-β catalyzed by P-glycoprotein
Source: PLoS One. 2021 Apr 26;16(4):e0250371. doi: 10.1371/journal.pone.0250371 (PMC8075256; doi:10.1371/journal.pone.0250371)
Supplement: S2 Table — (DOCX) [file pone.0250371.s011.docx]

**S2 Table. Movement of Aβ and Daunorubicin by P-gp According to Structural Transition.**

|  | **Start (4KSB)** | **Δ 4KSB -> 3B5X** | **3B5X** | **Δ 3B5X -> 2HYD** | **2HYD** | **Δ 2HYD- > 3B5Z** | **End (3B5Z)** |
| --- | --- | --- | --- | --- | --- | --- | --- |
| **Aβ 42** | 0.00 | -1.55 ± .054 | -1.55 ± .054 | -4.44 ± 1.68 | -5.98 ± 1.80 | -2.45 ± 1.06 | -8.44 ± 1.27 |
| **Aβ 40 2M4J** | 0.00 | -3.11 ± 0.67 | -3.11 ± 0.67 | -3.77 ± 0.96 | -6.88 ± 1.16 | -2.54 ± 0.74 | -9.42 ± 1.01 |
| **Aβ 40 2LFM** | 0.00 | -1.04 ± 0.98 | -1.04 ± 0.98 | -2.72 ± 1.88 | -3.76 ± 1.84 | -4.06 ± 2.76 | -7.83 ± 1.29 |
| **POLY42** | 0.00 | 5.11 ± 1.31 | 5.11 ± 1.31 | -3.01 ± 1.14 | 2.11 ± 2.22 | -1.89 ± 0.99 | 0.21 ± 2.58 |
| **DAU** | 0.00 | -2.85 ± 2.21 | -2.85 ± 2.21 | -2.70 ± 2.74 | -5.54 ± 3.41 | -4.59 ± 2.24 | -10.13 ± 2.72 |

Distances are reported in Angstroms relative to the center of mass coordinates at the start of the simulation, and are reported as mean ± standard deviation, n = 6.
