# Supplementary material for: Transport of Alzheimer’s associated amyloid-β catalyzed by P-glycoprotein
Source: PLoS One. 2021 Apr 26;16(4):e0250371. doi: 10.1371/journal.pone.0250371 (PMC8075256; doi:10.1371/journal.pone.0250371)
Supplement: S3 Table — (DOCX) [file pone.0250371.s012.docx]

**S3 Table. Mean Brightness of Aβ42 Fluorescence Intensity in Cell Culture Experiments**.

| **Cell Line** | **Treatment** | **Mean Fluorescence Intensity (a.u.) ± standard deviation** |
| --- | --- | --- |
| DU145 | 1µM Aβ | 17.65 ± 2.42 |
| DU145 | 1µM Aβ + 1µM TQR | 20.96 ± 1.62 |
| DU145-TXR | 1µM Aβ | 20.60 ± 1.62 |
| DU145-TXR | 1µM Aβ + 1µM TQR | 25.57 ± 2.38 |

The mean fluorescence intensity of paired chemotherapeutic sensitive/resistant cancer cell line (DU145 and DU145-TXR) as measured by confocal microscopy after incubation with 1 µM fluorescently labeled Aβ42 in the presence or absence of 1 µM Tariquidar (TQR). Statistical significance was determined using an unpaired T-test in Graphpad Prism; data are n = 24 images per treatment, two trials per treatment. Data are expressed as arbitrary units (a.u.) as calculated by the Integrated Density function of ImageJ [83-86]. The table shows the mean fluorescence intensity (a.u.) of per image ± one standard deviation from the mean.
