# Supplementary material for: Transport of Alzheimer’s associated amyloid-β catalyzed by P-glycoprotein
Source: PLoS One. 2021 Apr 26;16(4):e0250371. doi: 10.1371/journal.pone.0250371 (PMC8075256; doi:10.1371/journal.pone.0250371)
Supplement: Attachment — Submitted filename: Response_to_Reviewers.docx [file pone.0250371.s013.docx]

**Response to Reviewers:**

1. **The absolute rate of basal and maximal ATPase activity must be provided – i.e. in units of µmol nucleotide/min/mg protein (or µmol phosphate/min/mg protein).**

We have updated the methods to include this important information. In this study, the basal activity of P-gp in micelles was 51 ± 3 nmol/min/mg and the verapamil stimulated activity was 106 ± 7 nmol/min/mg; The basal activity of P-gp in nanodiscs was 131 ± 9 nmol/min/mg; and the verapamil stimulated activity was 390 ± 14 nmol/min/mg (n=4 technical replicates). It should be noted that one protein preparation was used for each of the different conditions (micelles and nanodiscs).

2. **What was the concentration of Aβ peptide used in ATPase assays. Only a molar ratio is provided.**

We have updated the methods to include this important information. The Aβ concentration used was 12.8 µM for P-gp in micelles, 9.6 µM for P-gp in nanodiscs. In micelles, 20 µg of purified P-gp was used; in nanodiscs, 15 µg of purified P-gp was used. Less protein in nanodiscs was used because it has a higher activity than protein in micelles. In micelles: 712nM P-gp to 12.8 µM Aβ42. In nanodiscs: 534 nM P-gp : 9.6 µM Aβ42. Both had a molar ratio of 1:18.

3. **The authors must indicate why specific concentrations of verapamil and Aβ peptide were used. The verapamil concentration of 150µM is exceedingly high and the conventional value used is in the range 10-30µM. Often higher concentrations of this compound can actually reduce the rate of ATP hydrolysis.**

A concentration of 150 µM verapamil was selected as this was the amount previously used to obtain maximal activity for murine P-gp expressed in *Pichia pastoris* as reported in Lerner-Marmarosh et al [1]. We agree that this concentration is high compared to different constructs and methods of expression. We measured the dependance of P-gp activity on different concentrations of verapamil and found that both methods of preparing P-glycoprotein tolerated high amounts of verapamil (Reviewer Figure 1).

**Reviewer Figure 1: Dose response of Murine P-gp to Verapamil.** Using a coupled enzyme assay, the hydrolysis of ATP to ADP by P-gp was coupled to the oxidation of NADH to NAD+. The activity of P-gp in either (A) micelles, or (B) nanodiscs was measured in increasing concentrations of Verapamil. Error bars and ± represent one standard error of the mean. Protein prep and kinetics assay was done as described in main text methods. For the Aβ peptide we selected a molar ratio of 1:18 P-gp to Aβ peptide, corresponding to roughly 10 µm Aβ42. 10 µm Aβ42 was selected as it was the highest concentration used in Bello et al. for similar experiments; additionally, cells can uptake and concentrate soluble Aβ42 into the low micromolar range [2, 3].

4. **A recent manuscript (New Evidence for P-gp-Mediated Export of Amyloid-β Peptides in Molecular, Blood-Brain Barrier and Neuronal Models. International Journal of Molecular Sciences. 2021; 22(1):246.) demonstrated a dose-dependent effect of two Aβ peptides on ATPase activity of Pgp in liposomes. The extent of their stimulation was considerably higher than in the current paper. Potentially it is a concentration issue. The earlier study should be cited, hydrolysis rates (in absolute values) compared and a reason for the discrepancy offered.**

In Chai et al. an increase in ATPase activity of about 1.5 fold was found for Aβ42 at a concentration of 10 µM [4]. Here we observed an insignificant increase in P-gp activity in micelles, and a fold-basal increase of 1.42 in nanodiscs. Given the different membrane environment, (vesicles vs nanodiscs) and protein type (human vs murine), we find the close agreement to be startling. The absolute basal and verapamil-stimulated activity of P-gp in our work is lower than that reported in Chai et al. (basal 131 vs 419 maximal 390 vs 1815) [4]. Given that the results of both studies are comparable to published values and rates of stimulation [1], we hypothesize that a major factor in activity differences is due both to the protein type and the expression system used (murine in yeast and human in insect cells). However, it should be noted that we each employed a different membrane system, and this could significantly affect the ATPase activity levels of P-gp. The paper here mentioned, Chai et al., was published after this paper had been submitted. We find this paper to be excellent and have included a discussion of its findings in relation to our own in the results of the ATPase section and in the discussion.

5. **On page 13, line 246 the authors suggest that Aβ peptide stimulates the ATPase activity of P-gp to a lesser extent than verapamil. That cannot be claimed when using a single concentration of each compound! At those two concentrations there is a difference, but one cannot say that the extent of stimulation will follow this order throughout a concentration range.**

We agree with this assessment by the reviewers and have updated the text accordingly. We have changed the sentence in question from: “However, we observed that the Aβ42-stimulated activity was less than half of the ATPase activity stimulated by verapamil (VPL).”

To: "However, at the tested concentrations, we observed that the Aβ42-stimulated activity was less than half of the ATPase activity stimulated by verapamil (VPL), but we note that this relationship may not extend across the entire possible concentration range.”

6. **The point that the ATPase activity is lower in micelles than membranes (i.e. nanodiscs here) is certainly not a new one. It may be argued that due to the rigid environment of a nanodisc, that the peptides will preferentially enter the micelle environment to stimulate P-gp activity. Intercalation in a micelle will be energetically more favourable than a bilayer for the peptide.**

We acknowledge this comment by the reviewers. However, we feel that if the peptides preferentially entered the micelle environment to stimulate P-gp activity, as suggested above, then the Aβ42-stimulated ATPase activity of P-gp in micelles would presumably be higher than that of P-gp in nanodiscs. Furthermore, we feel that the Aβ42 peptides, due to their hydrophobic nature, would preferentially associate with a lipid bilayer, but this remains to be proven. We also feel that any inherent rigidity of a lipid nanodisc would depend upon the size of the nanodisc used.

The difference between the activity and behavior of P-gp in nanodiscs and micelles is well documented [5-7]. Furthermore, the properties of P-gp in nanodiscs have been shown to be comparable to its properties in native membranes, particularly in regard to ATPase activity [5, 8, 9]. Therefore, with the current data available, we feel it is appropriate to say that there are differences in the behavior of P-gp when in variable lipid environments – e.g. nanodiscs, micelles, or liposomes.

7. **Why was murine P-gp chosen for the ATPase activity studies, considering that rodents express multiple isoforms ? And why was only 1-42 included in these experiments and 1-40 left out ?**

For the purposes of our experiments, we found that the murine P-gp is more stable when purified; consequently murine P-gp was used for our ATPase activity studies. The A and B isoforms of mouse P-gp (UniProtKB - P21447 (MDR1A_MOUSE), and UniProtKB - P06795 (MDR1B_MOUSE)) share an 87.33% or 80.56% sequence identity with human P-gp (UniProtKB - P08183 (MDR1_HUMAN)), respectively. We employed the MDR1A_MOUSE variant in these studies because of its superior stability relative to MDR1_HUMAN in these assays.

We chose Aβ42 for our microscopy and ATPase experiments because Aβ42, specifically Aβ42 oligomers, are associated with neurotoxic effects [10-12]. Furthermore, a previous cell culture studies focused on P-gp and Aβ42 produced conflicting results [2]. Similar to our study, Bello & Salerno used a paired P-gp overexpressing and non-overexpressing cell line - K562/K562-ADR P-gp overexpressing cells – but results were measured using membrane vesicles [2]. Additionally, the aforementioned study did not use a strong P-gp inhibitor, but a substrate (Pirarubicin) to test the effects of AB42. This approach has significant limitations, because Pirarubicin is a well known P-gp substrate, and evidence suggest that AB peptides are *weak* substrates of P-gp. Thus it is possible that Pirarubicin outcompeted the AB peptides for transport, leading Bello & Salerno to conclude that it is not a P-gp substrate. Lastly, We know that the activity of P-gp is strongly affected by the surrounding lipid environment, and similar to the contemporary paper by Chai et al. 2021, we sought to further explore the P-gp-AB42 relationship using a more native membrane environment (nanodiscs), and compare it to results in micelles [4].

8. **On Page 4, the authors compare the ATPase activity of P-gp reconstituted in micelles vs nanodiscs and conclude that the difference in findings is due to difference in membrane environment. Considering that they are comparing two different ‘in vitro’ methods, all with their pro’s and con’s as well as different incorporation efficiencies, this conclusion needs to be toned down as it is highly speculative and not based on much.**

We sincerely agree with this assessment by the reviewers and the text has been modified accordingly.

9. **PMID: 33022784; provided molecular docking data on the interaction of both Aβ40 and Aβ42 peptide with Pgp. How do those docking data compare with the MD simulations in this manuscript?**

We thank the reviewers for pointing us towards this excellent work, and have updated the text to include a brief discussion of these findings. Both our docking studies and those performed by Callaghan et al. suggest that Aβ peptides can fit within the transmembrane drug binding domains of P-gp [13]. In the 4m1m-corrected PDB structure of mouse P-gp (used by Callaghan et al), the transmembrane drug binding domain is less open than in the 4ksb PDB structure of mouse P-gp (used in our work). Therefore, even though the volume of the drug-binding cavity in 4m1m is less than that of 4ksb, the Aβ peptides are still able to fit within the cavity.

10. **Nosol et al. “Cryo-EM structures reveal distinct mechanisms of inhibition of the human multidrug transporter ABCB1.” Proc Natl Acad Sci U S A 117, 26245-26253 (2020) provides significant information on residues involved with substrate binding. Does the docking data in the current manuscript reconcile with** [**this reference (linked here)**](https://pubmed.ncbi.nlm.nih.gov/33020312/)**?**

We thank the reviewers for pointing us to this excellent work. In the study conducted by Nosol et. al, the P-gp substrate vincristine was found to interact with 13 residues within the drug binding domains of the transporter - M68, M69, Y953, F983, Y310, I306, M949, E875, M986, Q946, Q347, F343, and Q990; these interactions are contributed by TM11, TM1, TM12, TM6, TM10, and TM5 (Figure S7, Nosol et al.) [14]. In our docking studies of Aβ42 and Aβ40, for each of the three structures used, we find that the Aβ peptides are indeed interacting closely with 11/13 of the specified residues, the exceptions being F983 and M68.

Nosol et al. define these close contacts as being within 3.4 Å of vincristine, and we find that M68 is approximately 4 Å from the Aβ peptides. F983 is approximately 5 Å from the Aβ peptides. We find that the lack of close interactions (less than or equal to 3.4 Å) between Aβ peptides and F983 or M68 is easily explained by the difference in the conformation of the transporter. Nosol et al state that, *“[i]n contrast to the human–mouse chimeric ABCB1–UIC2-Fab data (25), the inward-open conformation (with straight TM4 and TM10) was not observed in our datasets. This suggests that binding of MRK16-Fab may shift the conformational equilibrium of lipid embedded ABCB1 from inward open to occluded.”* Thus in the conformations of P-gp solved by Nosoel et al, TM4 and TM10 are kinked, and the transporter is not in the inward-open conformation.

The shift in the orientations of TM4 and TM10 brings F983 and M68 closer to the substrate within the drug binding domain. Our TMD simulations and docking experiments both used P-gp in an inward-open conformation, as this corresponds to the first step in a putative transport cycle. Therefore, the orientation of helices TM4 and TM10 is such that F938 and M68 are slightly too far away from the Aβ peptides to facilitate close interactions. Nonetheless, our findings correlate well with those of Nosol et al., and support the hypothesis that these 13 residues are important for substrate binding.

11. **One of the key points raised in the earlier manuscript was the potential difficulty in a conventional alternating access model being able to move Aβ peptides. It was suggested that the peptides might be “pinched” during the transport cycle. The authors should comment on how they see the large peptide traversing the cavity in what is regarded as quite a tight fit.**

The ability of P-gp to move substrates of a large molecular weight is an open question. Interestingly, in our TMD simulations, we did observe that the conformational changes of the transporter resulted in a ‘pinching’, or ‘squeezing’, of the Aβ peptides throughout the simulated transport cycle. However, additional considerations arise from the difference between our docking study, and that performed by Callaghan et al [13].

Firstly, our docking studies used a model of human P-gp based upon the 4KSB structure of murine P-gp. In contrast, Callaghan et al. docked Aβ peptides against the 4M1M-corrected structure of murine P-gp. The cavity formed by the transmembrane Drug Binding Domains (DBDs) of the 4M1M-corrected structure is smaller than that of the 4KSB structure. Therefore, Aβ had ‘more room to move’ than in the 4M1M-corrected structure.

Secondly, and most notably, the docking experiments performed by Callaghan et al. produced structures of the Aβ peptides in which the helical structures were largely present in the final docking pose (Callaghan et al., Figure 2C-E). This difference seems to arise from the docking program used – CLUSPRO 2.0, whereas our experiments used *Autodock Vina* [15, 16]. In our docking experiments, the Aβ peptides were allowed to be significantly more flexible than in CLUSPRO 2.0, which performs rigid-body docking prior to RMSD optimization – “The protein-protein docking server ClusPro 2.0 performs three computational steps: (i) rigid body docking; (ii) RMSD based clustering of the 1000 lowest energy structures; and (iii) removal of steric clashes by energy minimization”.

In our TMD simulations, when Aβ is within the transmembrane helices of P-gp at the start of the simulated transport cycle, part of the protein is exposed to the solvent. Since we know that Aβ are highly dependent upon the environment, and that helical structures collapse in water [17], it is possible that Aβ could interact with P-gp in a more disordered state. This partial or complete ‘collapse’ of the ordered helices could facilitate the fit, and subsequent transport, of these peptides through the DBDs of P-gp. Indeed, we see in our TMD simulations that the Aβ peptides are ‘pinched’ during the transport process.

It has also been suggested that P-gp substrates bind directly from the bilayer milieu, or even directly to the central cavity [13]. Since Aβ peptides are thought to arrive at P-gp through a complex network of interactions, some even involving liposomes, it is possible that Aβ peptides would contact the solvent before entering the transmembrane DBDs, and thus would enter the transmembrane DBDs in a more disordered state.

12. **On page 9 (line 168) the authors claim that stimulation of ATPase activity renders a compound as a substrate. This does not hold true for many compounds - the text should be toned down.**

We agree with this assessment, especially in the case of P-gp inhibitors like Tariquidar, which both inhibits P-gp and stimulates the ATPase activity of the transporter. We have toned down the text. The lines in question are now #202-203, page 9.

Original line:

“The stimulation of ATPase activity by Aβ42 indicates that it interacts directly with P-gp, and supports the hypothesis that Aβ42 is a transport substrate of P-gp.”

Revised:

“The stimulation of ATPase activity by Aβ42 indicates that it *may* *interact* directly with P-gp, and supports the hypothesis that Aβ42 is a *putative* transport substrate of P-gp.”

13. **The fluorescent moiety is “Hylyte488-Aβ42” and this should replace the "fl-Aβ".**

We agree that the original method of referring to our fluorescently-labeled Aβ42 was confusing, and we thank the reviewers for suggesting this change. We have updated the text to include the specific reference to ‘Hilyte488-Aβ42” where appropriate.

14. **I cannot find the wavelengths used for the fluorescent analyses. Figure 4 contains a clue – GFP. Was the wavelength/filter set for this fluorophore.**

We thank the reviewers for pointing out these missing parameters and have added them to the manuscript. Fluorescence of Hilyte488-Aβ42 and of DAPI were measured using the pre-programmed EGFP or DAPI settings in the ZEN 3.3 software on the confocal microscope. Specifically, the EGFP filter uses excitation/emission wavelengths of 488/509 nm, and DAPI uses excitation/emission of 353/465 nm.

15. **The authors need to comment on why there is a Tariquidar effect on in the parental cell line (DU-145) in Figure 4 given that there is no Pgp? Tariquidar is known to affect ABCG2/BCRP – is this an explanation?**

We agree that these findings are puzzling. We feel it would be helpful to clarify that the non-P-gp-overexpressing, chemotherapy sensitive DU145 cells *do* express detectable levels of P-gp. In previous work performed by our group, using quantitative One-Step qPCR, we found that the chemotherapy-resistant DU145-TXR cells overexpress P‐gp, BCRP, and MRP1 by about 14,000‐fold, 4‐fold, and 0.75‐fold, respectively, in comparison to the chemotherapy-sensitive, parental DU145 cells (Holcomb-Webb et al. <https://doi.org/10.1096/fasebj.2018.32.1_supplement.531.19> and unpublished data from our group). Therefore, while the levels of expression are far below those of the DU145-TXR cell line, DU145 cells do express both P-gp and BCRP. This could explain the observed effect of Tariquidar upon the DU145 cells in our microscopy experiments.

In previous work performed by our group [18], we measured the intracellular accumulation of a fluorescent P-gp substrate in the presence or absence of P-gp inhibitors. While we would expect the uninhibited, P-gp overexpressing cells to exhibit the lowest level of fluorescence, we observed isolated puncta of fluorescence in these cell populations. Given the plasticity of cancer cell genetics, we postulated the existence of subpopulations within our MDR line that were not over-expressing P-gp to the expected level. It is possible that we are observing a similar but inverted effect here, in which some cells of the DU145 line are expressing more P-gp than others. This offers an additional explanation for the effect of TQR on the chemotherapy-sensitive line. We have included a discussion of these interesting results in the Discussion.

16. **On first glance, the error bars in Figure 4 do not suggest a significant difference. But the authors have used an apparent n=24, which facilitated reaching significance. That is a little disingenuous, since the data were actually taken from two separate sample preparations. The n=24 is essentially an internal replicate. The authors might consider sampling a minimum of three separate (or independent) sections/samples in order to provide a true value of the assay variability.**

We apologize for the lack of clarity in the statement about the trials and sample sizes in the microscopy experiments. We have updated the relevant portions of the text, and figures, to clarify any confusion.

17. **Figure 4 contains a single concentration of Aβ peptide (1µM). Why was this value chosen?**

For our experiments with Hylyte488-Aβ42 and Du145-TXR cells, we chose 1 µM for the following reasons – 1) this concentration provided detectable fluorescence of Hylyte488-Aβ42 without significant background fluorescence, and thus facilitated image quantification; 2) at this low concentration, we did not observe the formation of Hylyte488-Aβ42 aggregates or fibrils; and 3) pilot studies of this assay showed some toxicity effects at Hylyte488-Aβ42 concentrations greater than 5 µM. Furthermore, previous work by our group has focused upon the discovery and optimization of P-gp inhibitors, particularly inhibitors that are not P-gp substrates [18, 19]. In our assays with experimental compounds and Du145-TXR cells, we typically use concentrations in a range from 1 µM to 15 µM.

18. **The introduction section could, and should, be trimmed by one third.**

We have trimmed the introduction from ~850 words to ~650 words.

19. **Page 3, line 15 – the build-up of amyloid deposits is actually an imbalance in the relative extents of formation and clearance. It cannot be exclusively attributed to clearance.**

We thank the reviewers for pointing this out. We have changed the wording from:

“One of the pathological hallmarks of AD is deposition and accumulation of amyloid-β (Aβ) in the brain, which is thought to be caused by decreased clearance of Aβ from the brain.”

To: “One of the pathological hallmarks of AD is deposition and accumulation of amyloid-β (Aβ) in the brain, which is thought to be caused not only by increased Aβ production, but also by decreased clearance of Aβ from the brain.”

20. **Simply express p-values as <0.05, 0.01 etc.**

We have updated the relevant figures and instances within the text.

21. **In Figure 4a, the label comprises DU145 (Pgp wild type). label it as DU145 (sensitive) or (resistant).**

We have updated the figure appropriately.

22. **the apparent transport of Aβ peptide should be referred to as “simulated movement”**

Respectfully to the reviewer, we are concerned that the use of “simulated movement” may confuse the reader, primarily into believing that biasing forces (outside of the standard NAMD force fields) have been applied to the Aβ peptide(s). This is done in steered molecular dynamics simulations as in Moore et. al.; which, while a similar technique, produces profoundly different interpretations to the results, particularly because transport ligands are being pushed or pulled in a user-defined direction by external biasing forces [20]. We feel that we have taken sufficient care to present the results as a product of *in silico* experiments so as to prevent confusion over their origin, e.g. “although both structures of Aβ40 (2LFM and 2M4J) were moved from the inner to outer leaflet in our simulations...”.

However, we are sympathetic to the concern that when quantifying movement, it should not be phrased as transport. Especially as we have not defined cutoffs for how much movement is needed to be considered a transport substrate. We have changed ‘transport’ to ‘movement’ as in figure 2, which was titled: “Transport of Amyloid-β by P-glycoprotein gp in Molecular Dynamics Simulations” To: “Movement of Amyloid-β by P-glycoprotein gp in Molecular Dynamics Simulations”, and in other similar cases.

23. **The correct term (page 13 line 245) is dissociates and not “disassociates”**

We have replaced the occurrence(s) of ‘disassociates’ with ‘dissociates’.

24. **The authors have picked a polyarginine “control” peptide that is indeed similar in size and weight to 1-42 but very different in charge, therefore not being the best control here and not surprising that it wasn’t transported. Please comment.**

Respectfully, we disagree with the reviewer on this point. We selected the polyarginine control peptide (P42) to counter the concern that any substrate docked to the drug binding domains would be transported as an artifact of the simulation – specifically, an artifact of the targeted molecular dynamics techniques used. Furthermore, a contributing reason for the skepticism regarding Aβ as a transport substrate of P-gp, is that Aβ peptides are much larger than the commonly accepted substrates of P-gp [2]. Unfortunately, we were unable to identify any compounds of an equivalent size (to the Aβ peptides) which are also non-substrates of P-gp.

We considered using a scrambled Aβ peptide as the negative control for our TMD simulations. This might disrupt sequence-specific interactions, e.g., as with antibodies recognizing linear epitopes; however, if the mechanism of Aβ transport relies upon amino acid composition or other factors, we could reasonably expect that the scrambled peptide would be transported, and would therefore be a poor control. Given that the mechanism of substrate efflux by P-gp is still an area of active research, we were reluctant to assume that any one scrambled peptide would be an appropriate choice. With unlimited computational time and resources, multiple different types of scrambled and randomly mutated peptides would be ideal controls.

However, given the limitations in computing time, we chose P42, since its strong charge sets it apart from the typical characteristics of P-gp substrates, in a way that Aβ does not [21]. Given the promiscuity of P-gp and the lack of large, known non-substrates, we felt that P42, which is not at all expected to be transported by P-gp *in vitro*, to be an appropriate choice for our simulations.

25. **Why was 1-40 not included ?**

Due to the high cost of the peptide, we were limited to testing one isoform of Aβ for our experiments. We chose Aβ42 for our microscopy and ATPase experiments because Aβ42 is thought to have the most significant connection with Alzheimer’s disease, therefore it was the priority for our microscopy experiments [22, 23]. In prior research for this work, we also found that data on the relationship between P-gp and Aβ40 was more abundant than for Aβ42, and we sought to close this gap in the field [24].

26. **The images in Figure 4 are substandard and the authors need to provide better images.**

We have adjusted the contrast level of the merged images with the bright field channel so that the details of the cells are easier to see. We have also zoomed the images in significantly to facilitate better viewing. We have also included a supplementary movie containing Z-stacks of both DU145 and DU145-TXR cells, at 40X magnification, with and without Bright Field. The levels, contrast, and brightness of images were edited for publication using the following parameters in the Zen Blue 3.3 software – Bright field, level adjustment using the ‘Min-Max’ preset; DAPI (blue) – levels adjustment using the ‘Best-Fit’ setting; GFP green – level maxima set to 149. We have included the CZI source files for the Z-stacks as well. We want to reiterate that quantification methods were performed upon unaltered, unedited raw image files directly from the microscope. We have updated the methods accordingly to document these procedures in great detail, so that someone with the raw CZI files should be able to exactly replicate our images. Please let us know if any additional changes are needed.

27. **If the TXR cells in Figure 4 are overexpressing P-gp, wouldn’t one expect that they have much less 1-42 (i.e. GFP) signal as they keep the amyloid peptide out ? Why then is the GFP image for the DU145 vs DU145-TXR (untreated with the P-gp inhibitor) in both cases similar in signal quantity ?**

We agree that these results raise interesting questions. We observed the increased accumulation of Hylyte488-Aβ42 in the presence of Tariquidar in both P-gp overexpressing DU145-TXR cells and the parental, non-P-gp overexpressing DU145 cells (Figure 4). We also observed that the uninhibited DU145-TXR cells exhibited significantly higher fluorescence than the uninhibited DU145 cells. We hypothesize that this is due to reduced CD33 levels in the DU145-TXR cell line (3.75 fold decrease relative to DU145 cells), a byproduct of generating multidrug resistance through increasing exposure to Paclitaxel [25]. CD33 is a transmembrane protein and is involved in both cellular adhesion and Aβ clearance processes; interestingly, reduced expression of CD33 has been shown to correlate with increased uptake of Aβ42 [25-28]. Nonetheless, inhibition of the resistant DU145-TXR cells resulted in a 33% greater change in intracellular Aβ fluorescence relative to the change in the parental DU145 cells, again strongly suggesting that inhibition of P-gp transport resulted in low levels of Aβ efflux through P-gp, with a concomitant increase in Aβ accumulation.

28. **Also, a recent paper by Chai et al (IJMS Dec 2020) shows that both 1-40 and 1-42 peptides stimulate ATP hydrolysis of human P-gp. Please discuss the results in light of your own findings.**

We thank the reviewers for bringing these findings to our attention. The paper here mentioned, Chai et al., was published after this paper had been submitted. We find this paper to be excellent and have included a discussion of its findings in relation to our own in the results of the ATPase section and in the discussion.

**References**

1.	Lerner-Marmarosh N, Gimi K, Urbatsch IL, Gros P, Senior AE. Large scale purification of detergent-soluble P-glycoprotein from Pichia pastoris cells and characterization of nucleotide binding properties of wild-type, Walker A, and Walker B mutant proteins. J Biol Chem. 1999;274(49):34711-8. Epub 1999/11/27. doi: 10.1074/jbc.274.49.34711. PubMed PMID: 10574938.

2.	Bello I, Salerno M. Evidence against a role of P-glycoprotein in the clearance of the Alzheimer's disease Abeta1-42 peptides. Cell Stress Chaperones. 2015;20(3):421-30. doi: 10.1007/s12192-014-0566-8. PubMed PMID: 25591827; PubMed Central PMCID: PMCPMC4406933.

3.	Hu X, Crick SL, Bu G, Frieden C, Pappu RV, Lee JM. Amyloid seeds formed by cellular uptake, concentration, and aggregation of the amyloid-beta peptide. Proc Natl Acad Sci U S A. 2009;106(48):20324-9. Epub 2009/11/17. doi: 10.1073/pnas.0911281106. PubMed PMID: 19910533; PubMed Central PMCID: PMCPMC2787156.

4.	Chai AB, Hartz AMS, Gao X, Yang A, Callaghan R, Gelissen IC. New Evidence for P-gp-Mediated Export of Amyloid-beta PEPTIDES in Molecular, Blood-Brain Barrier and Neuronal Models. Int J Mol Sci. 2020;22(1). Epub 2021/01/02. doi: 10.3390/ijms22010246. PubMed PMID: 33383667; PubMed Central PMCID: PMCPMC7795149.

5.	Lusvarghi S, Ambudkar SV. ATP-dependent thermostabilization of human P-glycoprotein (ABCB1) is blocked by modulators. Biochem J. 2019;476(24):3737-50. Epub 2019/11/28. doi: 10.1042/BCJ20190736. PubMed PMID: 31774117; PubMed Central PMCID: PMCPMC7404625.

6.	Shukla S, Abel B, Chufan EE, Ambudkar SV. Effects of a detergent micelle environment on P-glycoprotein (ABCB1)-ligand interactions. J Biol Chem. 2017;292(17):7066-76. Epub 2017/03/12. doi: 10.1074/jbc.M116.771634. PubMed PMID: 28283574; PubMed Central PMCID: PMCPMC5409473.

7.	Lusvarghi S, Durell SR, Ambudkar SV. Does the ATP-bound EQ mutant reflect the pre- or post-ATP hydrolysis state in the catalytic cycle of human P-glycoprotein (ABCB1)? FEBS Lett. 2021. Epub 2021/02/07. doi: 10.1002/1873-3468.14054. PubMed PMID: 33547668.

8.	Ritchie TK, Kwon H, Atkins WM. Conformational analysis of human ATP-binding cassette transporter ABCB1 in lipid nanodiscs and inhibition by the antibodies MRK16 and UIC2. J Biol Chem. 2011;286(45):39489-96. Epub 2011/09/23. doi: 10.1074/jbc.M111.284554. PubMed PMID: 21937435; PubMed Central PMCID: PMCPMC3234772.

9.	Zoghbi ME, Mok L, Swartz DJ, Singh A, Fendley GA, Urbatsch IL, et al. Substrate-induced conformational changes in the nucleotide-binding domains of lipid bilayer-associated P-glycoprotein during ATP hydrolysis. J Biol Chem. 2017;292(50):20412-24. Epub 2017/10/12. doi: 10.1074/jbc.M117.814186. PubMed PMID: 29018094; PubMed Central PMCID: PMCPMC5733581.

10.	Sengupta U, Nilson AN, Kayed R. The Role of Amyloid-beta Oligomers in Toxicity, Propagation, and Immunotherapy. EBioMedicine. 2016;6:42-9. Epub 2016/05/24. doi: 10.1016/j.ebiom.2016.03.035. PubMed PMID: 27211547; PubMed Central PMCID: PMCPMC4856795.

11.	Verma M, Vats A, Taneja V. Toxic species in amyloid disorders: Oligomers or mature fibrils. Ann Indian Acad Neurol. 2015;18(2):138-45. Epub 2015/05/29. doi: 10.4103/0972-2327.144284. PubMed PMID: 26019408; PubMed Central PMCID: PMCPMC4445186.

12.	Dahlgren KN, Manelli AM, Stine WB, Jr., Baker LK, Krafft GA, LaDu MJ. Oligomeric and fibrillar species of amyloid-beta peptides differentially affect neuronal viability. J Biol Chem. 2002;277(35):32046-53. Epub 2002/06/12. doi: 10.1074/jbc.M201750200. PubMed PMID: 12058030.

13.	Callaghan R, Gelissen IC, George AM, Hartz AMS. Mamma Mia, P-glycoprotein binds again. FEBS Lett. 2020. Epub 2020/10/07. doi: 10.1002/1873-3468.13951. PubMed PMID: 33022784.

14.	Nosol K, Romane K, Irobalieva RN, Alam A, Kowal J, Fujita N, et al. Cryo-EM structures reveal distinct mechanisms of inhibition of the human multidrug transporter ABCB1. Proc Natl Acad Sci U S A. 2020;117(42):26245-53. Epub 2020/10/07. doi: 10.1073/pnas.2010264117. PubMed PMID: 33020312; PubMed Central PMCID: PMCPMC7585025.

15.	Porter KA, Xia B, Beglov D, Bohnuud T, Alam N, Schueler-Furman O, et al. ClusPro PeptiDock: efficient global docking of peptide recognition motifs using FFT. Bioinformatics. 2017;33(20):3299-301. Epub 2017/04/22. doi: 10.1093/bioinformatics/btx216. PubMed PMID: 28430871; PubMed Central PMCID: PMCPMC5860028.

16.	Trott O, Olson AJ. AutoDock Vina: improving the speed and accuracy of docking with a new scoring function, efficient optimization, and multithreading. J Comput Chem. 2010;31(2):455-61. doi: 10.1002/jcc.21334. PubMed PMID: 19499576; PubMed Central PMCID: PMCPMC3041641.

17.	Yang M, Teplow DB. Amyloid beta-protein monomer folding: free-energy surfaces reveal alloform-specific differences. J Mol Biol. 2008;384(2):450-64. Epub 2008/10/07. doi: 10.1016/j.jmb.2008.09.039. PubMed PMID: 18835397; PubMed Central PMCID: PMCPMC2673916.

18.	Nanayakkara AK, Follit CA, Chen G, Williams NS, Vogel PD, Wise JG. Targeted inhibitors of P-glycoprotein increase chemotherapeutic-induced mortality of multidrug resistant tumor cells. Sci Rep. 2018;8(1):967. Epub 2018/01/19. doi: 10.1038/s41598-018-19325-x. PubMed PMID: 29343829; PubMed Central PMCID: PMCPMC5772368.

19.	Nanayakkara AK, Vogel PD, Wise JG. Prolonged inhibition of P-glycoprotein after exposure to chemotherapeutics increases cell mortality in multidrug resistant cultured cancer cells. PLoS One. 2019;14(6):e0217940. Epub 2019/06/08. doi: 10.1371/journal.pone.0217940. PubMed PMID: 31173617; PubMed Central PMCID: PMCPMC6555590.

20.	Moore DS, Brines C, Jewhurst H, Dalton JP, Tikhonova IG. Steered molecular dynamics simulations reveal critical residues for (un)binding of substrates, inhibitors and a product to the malarial M1 aminopeptidase. PLoS Comput Biol. 2018;14(10):e1006525. Epub 2018/11/01. doi: 10.1371/journal.pcbi.1006525. PubMed PMID: 30379805; PubMed Central PMCID: PMCPMC6239339.

21.	Schinkel AH, Jonker JW. Mammalian drug efflux transporters of the ATP binding cassette (ABC) family: an overview. Adv Drug Deliv Rev. 2003;55(1):3-29. Epub 2003/01/22. doi: S0169409X02001692 [pii]. PubMed PMID: 12535572.

22.	Naslund J, Schierhorn A, Hellman U, Lannfelt L, Roses AD, Tjernberg LO, et al. Relative abundance of Alzheimer A beta amyloid peptide variants in Alzheimer disease and normal aging. Proc Natl Acad Sci U S A. 1994;91(18):8378-82. Epub 1994/08/30. doi: 10.1073/pnas.91.18.8378. PubMed PMID: 8078890; PubMed Central PMCID: PMCPMC44609.

23.	Mori H, Takio K, Ogawara M, Selkoe DJ. Mass spectrometry of purified amyloid beta protein in Alzheimer's disease. J Biol Chem. 1992;267(24):17082-6. Epub 1992/08/25. PubMed PMID: 1512246.

24.	Chai AB, Leung GKF, Callaghan R, Gelissen IC. P-glycoprotein: a role in the export of amyloid-beta in Alzheimer's disease? FEBS J. 2020;287(4):612-25. Epub 2019/11/22. doi: 10.1111/febs.15148. PubMed PMID: 31750987.

25.	Takeda M, Mizokami A, Mamiya K, Li YQ, Zhang J, Keller ET, et al. The establishment of two paclitaxel-resistant prostate cancer cell lines and the mechanisms of paclitaxel resistance with two cell lines. Prostate. 2007;67(9):955-67. Epub 2007/04/19. doi: 10.1002/pros.20581. PubMed PMID: 17440963.

26.	Griciuc A, Serrano-Pozo A, Parrado AR, Lesinski AN, Asselin CN, Mullin K, et al. Alzheimer's disease risk gene CD33 inhibits microglial uptake of amyloid beta. Neuron. 2013;78(4):631-43. Epub 2013/04/30. doi: 10.1016/j.neuron.2013.04.014. PubMed PMID: 23623698; PubMed Central PMCID: PMCPMC3706457.

27.	Bradshaw EM, Chibnik LB, Keenan BT, Ottoboni L, Raj T, Tang A, et al. CD33 Alzheimer's disease locus: altered monocyte function and amyloid biology. Nat Neurosci. 2013;16(7):848-50. Epub 2013/05/28. doi: 10.1038/nn.3435. PubMed PMID: 23708142; PubMed Central PMCID: PMCPMC3703870.

28.	Zhao L. CD33 in Alzheimer's Disease - Biology, Pathogenesis, and Therapeutics: A Mini-Review. Gerontology. 2019;65(4):323-31. Epub 2018/12/13. doi: 10.1159/000492596. PubMed PMID: 30541012.
