# Supplementary material for: Transport of Alzheimer’s associated amyloid-β catalyzed by P-glycoprotein
Source: PLoS One. 2021 Apr 26;16(4):e0250371. doi: 10.1371/journal.pone.0250371 (PMC8075256; doi:10.1371/journal.pone.0250371)
Supplement: Attachment — Submitted filename: Response to Reviewers2.docx [file pone.0250371.s014.docx]

**Reviewer #1: The revised article has addressed the vast majority of the points raised in the first round. It has been substantially improved; however, there are some minor adjustments that are needed as indicated below.**

**Reviewer #2 – all issues were addressed**

**Point 1 (ATPase activity): The values for ATP hydrolysis are a little on the low side, but within the acceptable range. The authors provided the absolute values in the revised version but have done so in the methods section. These are quite clearly results and should be placed in the section of results dealing with ATP hydrolysis.**

We have updated the results section on page 8 to include the following statement:

**“**In micelles, we report that the basal activity of P-gp was 51 ± 3 nmol/min/mg, and the VPL-stimulated activity was 106 ± 7 nmol/min/mg. In nanodiscs, we report that the basal activity of P-gp was 131 ± 9 nmol/min/mg, and the VPL-stimulated activity was 390 ± 14 nmol/min/mg.”

**Point 2 (peptide concentrations): The molar concentrations should be added to the legend for Figure 3 and so should the values for verapamil.**

We have updated the legend of Figure 3 to include this important information. The following sentences were added in response to this request:

“For P-gp in micelles, 12.8 µM Aβ42 and 712 nM P-gp were used; for P-gp in nanodiscs, 9.6 µM Aβ42 and 534 nM P-gp were used – both corresponding to a molar ratio of 1:18. A VPL concentration of 150 µM was used, as in [51].”

**Point 3 (high verapamil concentrations)- The first sentence of the rebuttal (and the citation to Lerner-Marmarosh) should be added to the results section on ATP hydrolysis.**

We agree with this suggestion, and we have added the sentence – and accompanying citation - to the ATPase activity of P-gp in the presence of AB42 section of Results (page 8):

“A concentration of 150 µM verapamil was selected as this was the amount previously used to obtain maximal activity for murine P-gp expressed in *Pichia pastoris* as reported in Lerner-Marmarosh et al [49].”

**Point 7 (using only peptide 1-42)- The justification is acceptable and I would recommend adding the statement on the use of 1-42 peptide since it is the more neurotoxic should be added to the manuscript – potentially in the methods or results section.**

We agree with this suggestion. We have added the following statement under the “preparation of the AB42 synthetic peptide” section in Methods; references correspond to the bibliography:

“Previous cell culture studies of P-gp and Aβ42 produced conflicting results [12]. Additionally, Aβ42, specifically the oligomeric form, is associated with neurotoxic effects [76-78]. Thus, we sought to further explore, and hopefully elucidate, the relationship between P-gp and Aβ42 in our cell-based and biophysical assays.

**Point 11 (size of peptide)- The author’s rebuttal is well argued and raises many important points. The issue of substrate size and the perceived inability of P-gp to transport the peptides is a common argument in the field. It is used frequently to dismiss the involvement of P-gp in amyloid peptide removal – despite the considerable evidence. Consequently, I would advise an abbreviated version of this rebuttal be inserted into the manuscript. This would provide significant added value to the community.**

**Original comment (for our reference): “One of the key points raised in the earlier manuscript was the potential difficulty in a conventional alternating access model being able to move Aβ peptides. It was suggested that the peptides might be “pinched” during the transport cycle. The authors should comment on how they see the large peptide traversing the cavity in what is regarded as quite a tight fit.**

We agree with this suggestion. We have inserted an abbreviated version of our rebuttal into the appropriate sections of the discussion. Below, we have copied the original discussion text, and then the revised discussion text below. Text in *italics* was inserted or changed in response to this request from our reviewers. We feel that these additions significantly enhance the discussion and fully cover the requested changes. We note that we also discuss the docking results in the Results section on p 5-6, lines 108-117. Full changes are tracked on our re-submitted version of the paper.

**Original Discussion (p11-12) ----------------------------------------**

The mechanism by which P-gp might transport substrates of such significant size and flexibility as the Aβ peptides remains unclear. Despite being particularly suited to exploring problems of this nature, MD simulations of AB and P-gp have not been performed. Using previously established techniques, we explored how P-gp might transport Aβ40 and Aβ42 using targeted MD simulations.

In each simulation of the Aβ peptides, we observed vectorial movement of Aβ through the P-gp DBDs and towards the extracellular space, with total movement ranging between 7.8 and 9.4 Å (Figs 2 and S3). These distances correlate well with previously published movements of the P-gp substrate daunorubicin (DAU) in TMD simulations [35]. Interestingly, for both Aβ42 and the 2M4J structure of Aβ40, the bulk of observed movement occurred during the transition between the 3B5X and 2HYD conformations of P-gp, or when the DBDs switch from open-to-the-inside to open-to-the-outside (S1 Fig, S1 Table). In contrast, for both DAU and the 2LFM structure of Aβ40, the bulk of observed movement occurred during the transition from 2HYD to 3B5Z, both of which are open-to-the-outside conformations (S1 Table).

Studies have shown that Aβ monomers can fold into structures with two β strands; these β strands allow the monomers…[Discussion section continues].

**Edited Discussion (p11-12) --------------------------------------**

The mechanism by which P-gp might transport substrates of such significant size and flexibility as the Aβ peptides remains unclear. *Specifically, two primary questions remain: firstly, can the Aβ peptides fit within the DBDs of P-gp at all, and secondly, are the alternating access motions of P-gp sufficient to move the Aβ peptides across the cell membrane [8]? In answer to the first open question, docking studies performed by us and by Callaghan et al. show that the DBDs of P-gp can accommodate the Aβ peptides in both ordered and disordered states, thus the initial association of Aβ with the DBDs should be possible [42]. To address the second question,* we explored how P-gp might transport Aβ40 and Aβ42 using targeted MD simulations *and previously established techniques*. *Our TMD simulations showed that P-gp is indeed capable of transporting the Aβ peptides through the membrane despite their significant sizes.*

In each simulation of the Aβ peptides, we observed vectorial movement of Aβ through the P-gp DBDs and towards the extracellular space, with total movement ranging between 7.8 and 9.4 Å (Figs 2 and S3). These distances correlate well with previously published movements of the P-gp substrate daunorubicin (DAU) in TMD simulations [35]. Interestingly, for both Aβ42 and the 2M4J structure of Aβ40, the bulk of observed movement occurred during the transition between the 3B5X and 2HYD conformations of P-gp, or when the DBDs switch from open-to-the-inside to open-to-the-outside (S1 Fig, S1 Table). In contrast, for both DAU and the 2LFM structure of Aβ40, the bulk of observed movement occurred during the transition from 2HYD to 3B5Z, both of which are open-to-the-outside conformations (S1 Table).

*As postulated by Callaghan et al., we observed that the Aβ peptides were indeed ‘pinched’ during simulated transport by P-gp [42]. However, in our TMD simulations, we found that the transported Aβ peptides were in a more disordered state than that postulated by Callaghan et al. This is unsurprising, since Aβ is partially exposed to the solvent when bound to the inward-open conformation of P-gp, and the helical structures of Aβ have been shown to collapse in water [54]. Since the Aβ peptides are thought to associate with P-gp though a complex network of interactions [8, 20, 55], it is possible that the peptides would contact the solvent prior to entering the DBDs of P-gp, and therefore arrive at the pump in a more disordered state. The partial – or complete – collapse of the ordered helices could further enhance the fit and subsequent transport of these bulky Aβ peptides by P-gp.*

Studies have shown that Aβ monomers can fold into structures with two β strands; these β strands allow the monomers…[Discussion section continues]

**Point 17 (fluorescent peptide concentration)**: **The justification for using 1uM fluorescent peptide is well made – please insert a statement in the methods section of the manuscript.**

We thank the reviewers for their assessment of our methods. We have inserted the following statement in the Accumulation of fluorescently labeled Aβ42 in DU145 and DU145-TXR Cells section of Methods (Page 19):

“We tested HiLyte-488-Aβ42 at a concentration of 1 µM for the following reasons: 1) at this concentration, HiLyte-488-Aβ42 was detectable without significant background fluorescence that would obfuscate quantification efforts; 2) the formation of HiLyte-488-Aβ42 fibrils was not observed at this concentration, and 3) our pilot studies observed toxicity effects against DU145-TXR cells at concentrations greater than 5 µM.”
